# Everyone matters: h indices as new metrics for educational evaluation

**DOI:** 10.3389/fpsyg.2025.1706451

**Published:** 2026-01-12

**Authors:** Nai-Ming Hou, Lu Xu

**Affiliations:** 1 School of Humanities, Tongren Polytechnic University, Tongren, China; 2 School of Sports and Health Science, Tongren University, Tongren, China

**Keywords:** educational equity, educational evaluation, h efficiency index, h index, h loss index

## Abstract

**Introduction:**

This paper addresses the limitations associated with using average scores for group evaluations. Drawing inspiration from the h-index in bibliometrics, we propose two new metrics for educational assessment: the h-efficiency index (HEI) and the h-loss index (HLI).

**Methods:**

The HEI is defined such that among the scores of *n* subjects, if at most E% of all scores are at least E% of the maximum possible score, then the value of HEI is E%. In contrast, the HLI is defined so that among the scores of n subjects, if at most L% of all scores are no more than (100-L)% of the maximum score, then the value of HLI is L%. This paper employs course examination results as a case study to compare the evaluation outcomes of HEI and HLI with those of average scores and medians.

**Results:**

The findings demonstrate that HEI and HLI are straightforward to compute and serve as complementary metrics. They take into account not only the overall performance level but also the distribution of scores among the subjects. Compared to average and median scores, HEI and HLI provide richer and more informative data for comprehensive evaluations.

**Discussion:**

HEI emphasizes high-scoring groups, while HLI focuses on low-scoring groups, reflecting statistical robustness and aligning with the measurement needs of educational equality and equity. It is noteworthy that, although this analysis centers on examination results, the applicability of HEI and HLI extends to a broader range of contexts. These metrics can effectively measure any evaluation items with continuous scores and defined maximum and minimum values, providing a concise and efficient evaluation tool.

## 1 Introduction

The thought of educational equity and educational equality has a long history, which can be traced back to Plato and Aristotle (Aydin et al., 2010; Howe, 2015; Heyneman, 2004). They are also basic principles widely accepted by modern human society and educational fields (Brookover and Lezotte, 1981; Calvert, 2014; Dadon-Golan et al., 2019; Hansen and Gustafsson, 2018; Kyriakides and Creemers, 2017; Schleicher and Zoido, 2016). There are three levels of educational equity (Crosby and Clayton, 2004; Roche, 2013; Summers and Smith, 2014): (1) everyone should enjoy equal rights and obligations to education; (2) relatively equal opportunities and conditions should be provided for education; and (3) everyone should have the relative equality of educational success opportunities and educational effects, that is, each student can reach the most basic standard after receiving the same level of education, including substantive fairness in students' academic performance, fairness in educational quality and equality in goal level. The meaning of modern educational equality mainly includes but is not limited to (Brando, 2017; Brighouse, 1995; Howe, 1993; Roche, 2013): (1) man is the purpose. The ultimate goal of man's education is the free and harmonious development of individuals. Only by respecting the development of each individual's basic human rights and freedom can it comply with the principle of educational equality; (2) the principle of equal rights in education; and (3) the principle of equal educational opportunities. A good education system means that everyone has equal access to school and equal opportunities to be treated and succeed in the process of education; and (4) the principle of differential treatment.

Based on the above principles of educational equality and educational equity, educational evaluation should consider, emphasize, and evaluate the performance of each individual and give appropriate adjustment and feedback on educational decisions and management (BenDavid-Hadar, 2016; Chaudhary et al., 2020; Crouch et al., 2020; Diaz et al., 2020; Hursh, 2005; LaVelle et al., 2020; Poth et al., 2020; Veletsianos, 2021). Educational evaluation should also reflect the overall performance of the research objects as much as possible (King and Ayoo, 2020), especially the individuals with relatively poorer performances; otherwise, it will lead to evaluation deviation and even decision-making mistakes. In education evaluation, it is often necessary to measure the score data of certain items to evaluate the performance of a group of research objects as the basis for in-depth analysis of data and decision-making. The most commonly used measurement metric is the arithmetic average, which is often used to reflect the general level or centralized trend of data. However, because high and low values offset each other, the average easily masks or ignores the performance of individuals with high or low scores and sometimes cannot sufficiently reflect the overall performance, resulting in the deviation or violation of the basic principles of educational equality and educational equity. In addition, the classification method according to the score (such as the classification of excellent, good, qualified, and unqualified) is also a common method. Although the classification comprehensively reflects the distribution of score data, it is not conducive to directly comparing the results of different groups.

As a new index in bibliometrics, in recent years, the h index has been used to evaluate individual or group academic achievements and influence (Hirsch, 2005) and has achieved great success worldwide (Alonso et al., 2009; Barnes, 2017). The advantage of the h index is that it uses a simple index to include both the number and frequency of highly cited papers. Based on the above considerations, this study proposes two new measurement metrics for education evaluation based on the mathematical idea of the h index. To meet the requirements of educational equity and equality as much as possible and pay special attention to objects with lower scores, the new metrics not only reflect the overall score level but also reflect the number of objects covered by the score level as much as possible. In the section of theory and methods, the definition of the new metrics and a brief comment on their meaning are given, and the data are briefly introduced; in the section of results and discussion, the data are evaluated and analyzed with the new metrics, mean and median, respectively, in order to compare the advantages and disadvantages of different methods. Finally, the nature and potential use of the new metrics are summarized, and conclusions are drawn for the paper.

## 2 Theory and methods

### 2.1 Definition of h-efficiency index

The definition of the h-efficiency index (HEI) is as follows:

*Among the scores of n objects, if at most E% of all the n scores is no less than E% of the full score, the value of HEI will be E%*.

The following example illustrates the calculation and significance of HEI. Taking students' course examination scores in a class as an example, suppose that the full score is 100 points; if at most 60% of the n students' scores are no less than 60 points, the value of HEI will be 0.60; if at most 90% of students' scores are no less than 90 points, the HEI value is 0.90; and so on. Obviously, the possible range of HEIs is between 0 and 1. The higher the overall scores, the higher the HEI value. Moreover, to obtain a higher HEI value, more students have to obtain a higher score. In contrast, even if only a few students achieve low scores (the degree of their influence is related to their proportion in the whole), their scores will be reflected in HEI. Therefore, the value of HEI considers not only the score level but also the number of students with high scores.

### 2.2 Definition of h-loss index

The definition of the h-loss index (HLI) proposed in this study is as follows:

*Among the scores of n objects, if the score of at most L% of the objects is not higher than (100-L) % of the full score, the value of the h-loss index will be L%*.

From the definition, it is obvious that HLI = 1-HEI. The following examples demonstrate the calculation and implications of HLI. Still take students' course examination scores in a class as an example. Suppose that the full score is 100 points; if at most 40% of the n students' scores are no more than 60 points, the value of HLI will be 0.40, which roughly corresponds to the situation in which 40% of students fail in the test (<60 points) (the objects whose scores are exactly equal to 60 points are ignored, which is trivial here). For a general course examination, the HLI was 0.40, and 40% of the students failed, which was obviously not a satisfactory result. If at most 20% of students' scores are no more than 80 points, the value of HLI will be 0.20, which corresponds to a much better situation than the former in terms of educational evaluation. If the HLI reaches 0.10, although it seems to be a relatively small value, it still reflects the need and space for improvement in teaching and learning. If one wants to further reduce the value of HLI, it obviously needs to make greater efforts, because more students must get 90 points or even higher. Obviously, the value range of HLI is also between 0 and 1. The lower the overall score, the greater the value of HLI. Moreover, to obtain a smaller HLI value, more students must obtain a higher score. HLI emphasizes the heterogeneity of learning outcomes, focusing on the breadth and depth of students' scores, especially the proportion of students with low scores. The close relationship between HLI and HEI reflects the diversity of educational achievements. The failure rate mainly focuses on the number of students who fail to meet the minimum requirements. It is a relatively simple evaluation method and focuses on the classification of qualified and unqualified students. In a sense, HEI and HLI are complementary to each other. They focus on the number of individuals with lower or higher scores in the whole. What they have in common is that they both consider the score level and the number of objects covered by the score level simultaneously. The proportions of objects with high scores and low scores are reflected in the two metrics.

### 2.3 Data and software

To compare different metrics, this paper uses the examination scores of the course of organic chemistry of two parallel classes, for which the full score is 100 points. The statistical analysis and calculation involved in this study were run on the MATLAB 7.0.1 platform (MathWorks, Sherborn, MA, USA) using self-compiled MATLAB code. The MATLAB code for calculating HEI and HLI could be requested from the corresponding author.

## 3 Results and discussion

**Figure 1:**
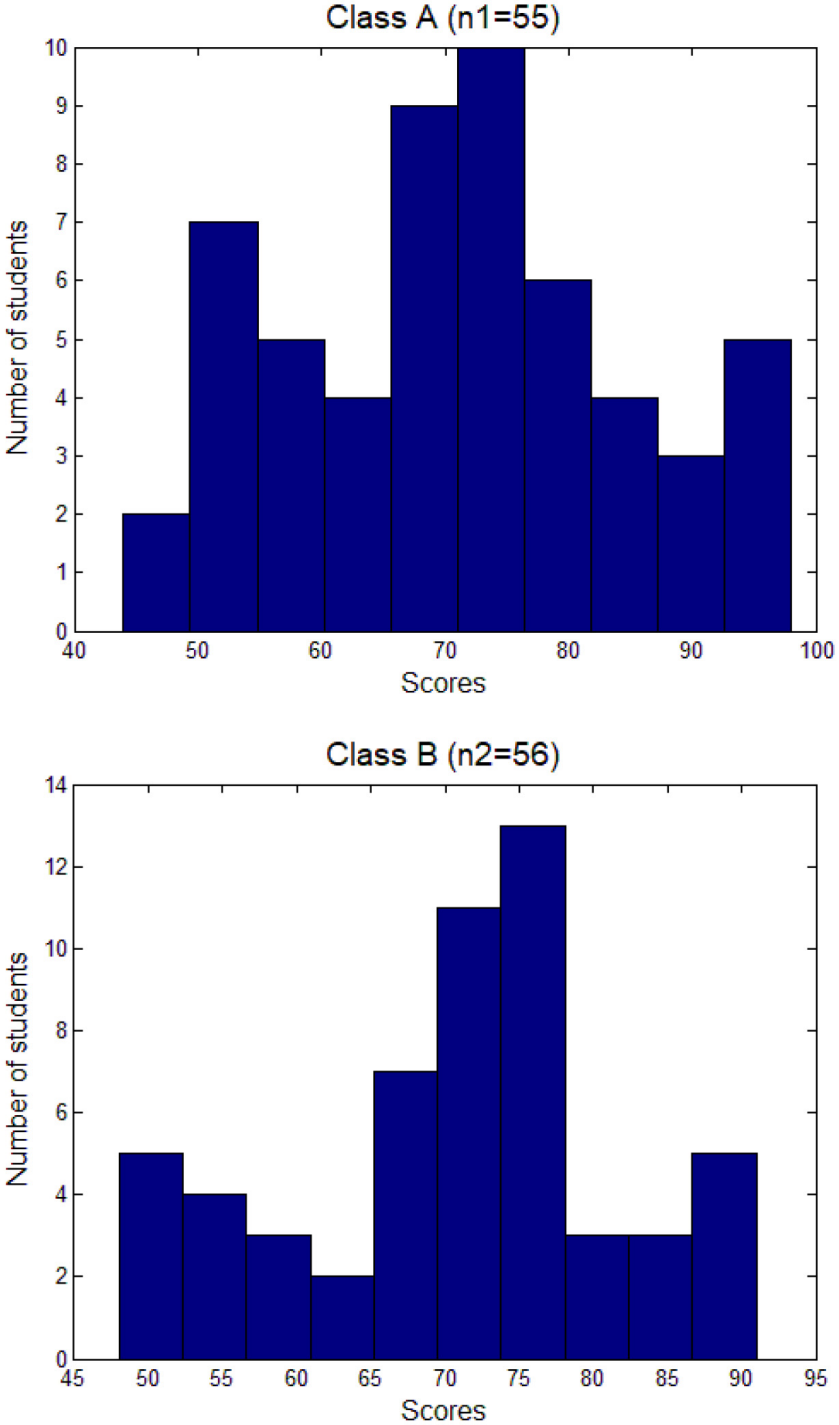
Histograms of scores of two classes in course examination of organic chemistry.

The original examination score data utilized in this study is presented in Table 1, while the results of the index calculations are shown in Table 2. Class A comprised 55 students, while Class B had 56 students. The average scores were 70.98 for Class A and 70.36 for Class B, with median scores of 72.00 and 71.00, respectively. The Shapiro-Wilk test indicated normality (*P* = 0.451), and the Durbin-Watson test assessed independence (*P* = 0.531). Although the *t*-test revealed no significant difference between the two averages (*P* = 0.796), Class A exhibited higher overall performance in terms of both average and median scores. Furthermore, an examination of the standard deviation and range of the data reveals that Class B (SD = 13.46) has a greater dispersion compared to Class A (SD = 11.40), a trend corroborated by the data range and histograms depicted in Figure 1.

**Table 1:** Examination scores of two classes in the course of organic chemistry.

**Classes**	**Scores (full score** = **100)**							
Class A (*n*1 = 55)	97	57	71	72	98	73	44	70
	54	73	53	83	75	53	55	77
	84	71	82	70	84	73	78	75
	61	70	64	54	62	75	57	74
	88	75	54	78	62	79	69	67
	50	66	47	77	91	58	74	93
	91	77	69	93	97	58	52	
Class B (*n*2 = 56)	91	77	66	87	54	71	69	68
	57	76	48	55	84	70	74	63
	73	71	79	77	61	75	52	79
	71	66	75	49	50	66	70	77
	70	86	71	48	91	55	77	71
	75	87	73	89	57	78	75	53
	66	84	77	77	73	57	67	82

**Table 2:** Comparison of basic metrics of scores of two classes in course examination of organic chemistry.

**Classes**	**Average**	**Median**	**SD**	**Highest**	**Lowest**	**HEI**	**HLI**
Class A (n1 = 55)	70.98	72.00	13.66	98.00	44.00	0.6610	0.3390
Class B (n2 = 56)	70.36	71.00	11.40	91.00	48.00	0.6698	0.3302

**Figure 2:**
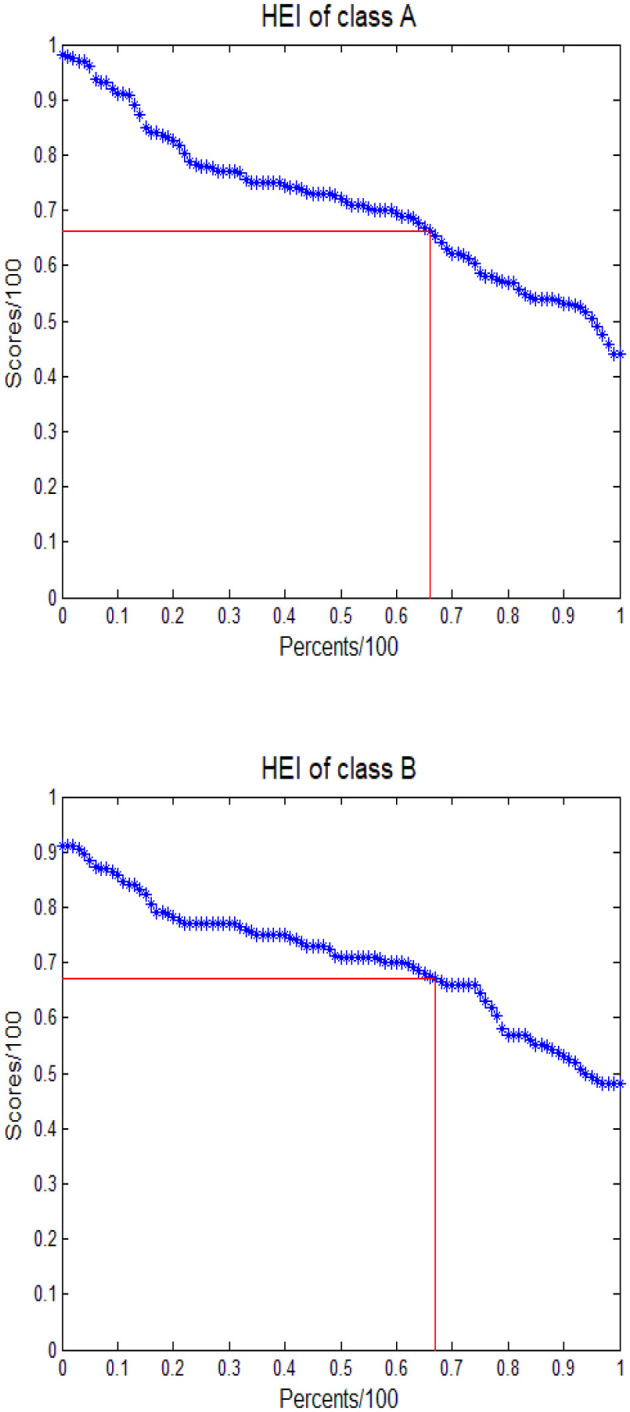
Calculation of HEI for the two classes in course examination of organic chemistry. HEI is the side length of the largest square contained under the curve of percentiles of scores (/100) vs. percent of students.

In Figure 2, we illustrate the calculations and geometric interpretations of the Higher Education Index (HEI). To construct the scatter plot, we calculated the quantiles of all subjects, using percentiles as the horizontal axis and their corresponding quantiles (divided by 100) as the vertical axis. The HEI represents the side length of the largest square that can be inscribed under the curve formed by the scatter points. For Class A, the HEI value is 0.6610, indicating that 66.10% of students scored at or above this threshold. The median score of 72.00 signifies that half of the scores are above this value, while the average score of 70.98 illustrates that the average can obscure distribution details due to the offsetting nature of high and low scores.

When comparing HEI, median, and average scores, HEI emphasizes the importance of both high scores and the number of students achieving them, thus demonstrating statistical robustness. As sample sizes increase, extreme scores have a diminished impact on HEI calculations. In contrast, the median offers a robust evaluation of overall scores, while the average provides the least information about score distributions. Furthermore, the Lower Education Index (LEI) can be calculated as 1–HEI, serving as a complementary measure to HEI that reflects the proportion of students with low scores.

It is essential to note that the focus of this data analysis is not to compare the scores of Class A and Class B directly. The differences in index values between the two classes are not statistically significant. Nonetheless, the results indicate that HEI and LEI can be effective tools for intuitively comparing groups. In this context, Class A (HEI = 0.6610) demonstrates slightly inferior performance compared to Class B (HEI = 0.6698), despite Class A's marginally higher average score (70.98 vs. 70.36). The calculations of HEI and LEI consider overall scores as well as the proportions of high and low scores, aligning with principles of educational equity and equality.

A comprehensive evaluation of individual performance is crucial in educational assessments, ensuring that adjustments and feedback in decision-making processes are well-informed. Moreover, HEI and LEI are statistically more reliable than average scores. Importantly, these indices can also be applied to assess educational outcomes beyond examination scores, as long as the scores are continuous and have clearly defined highest and lowest values. In conclusion, this study underscores the need for educational assessments to focus on equity-oriented metrics that consider the distribution of scores among students. By integrating HEI and LEI into evaluation frameworks, educational stakeholders can gain deeper insights into student performance, ultimately fostering a more equitable learning environment.

## 4 Conclusions

Taking the average is a common and basic measurement metric in educational evaluation. However, due to the mutual offset of high and low scores, the average tends to mask or ignore the performance of individuals with high or low scores and sometimes cannot sufficiently reflect the overall performance, resulting in the deviation or violation of the basic principles of educational equality and educational equity. This paper proposes two new measurement indices for educational evaluation, the h efficiency index (HEI) and the h loss index (HLI). Taking the overall evaluation of course examination results as an example, this paper compares the evaluation results of HEI, HLI, average score and median. The results show that HEI and HLI are complementary to each other. HEI and HLI consider not only the level of score but also the number of objects covered by the score level, which provides a richer amount of information for the overall evaluation. HEI and HLI are concise, practical, and statistically robust, which would meet the requirements of educational evaluation for educational equality and equity. Although this paper takes the measurement of examination results as an example, the application scope of the HEI and HLI should not be limited to the analysis of students' examination results but may also be applicable to a variety of other educational evaluation items.

## Data Availability

The raw data supporting the conclusions of this article will be made available by the authors, without undue reservation.
