# Supplementary material for: Everyone matters: h indices as new metrics for educational evaluation
Source: Front Psychol. 2026 Jan 12;16:1706451. doi: 10.3389/fpsyg.2025.1706451 (PMC12832614; doi:10.3389/fpsyg.2025.1706451)
Supplement: Supplementary file 1 [file Supplementary_file_1.docx]

**Supplementary file 1: MATLAB codes to compute HEI**

function [HEI]= hei(x)

%%% x is a set of evaluation data, such as the scores of a class, which is

%%% input in the form of row vector or column vector.

hv=zeros(1001,1);

tt=1-[1000:-1:0]'/1000;

for iii=0:1:1000

hv(iii+1)=prctile(x,(1000-iii)/10)/100;

end

[I0,I1]=min((abs(tt-hv)));

HEI=min(tt(I1),hv(I1));

figure

plot(tt(1:10:end),hv(1:10:end),'*')

hold on

plot([0, HEI],[ HEI, HEI])

hold on

plot([ HEI, HEI],[0, HEI])
